# The european paediatric legislation: benefits and perspectives

**DOI:** 10.1186/1824-7288-36-56

**Published:** 2010-08-17

**Authors:** Francesca Rocchi, Paolo Paolucci, Adriana Ceci, Paolo Rossi

**Affiliations:** 1 Ufficio Informazione e Comunicazione - Agenzia Italiana del Farmaco (AIFA), Via del Tritone, 181 - 00187 Roma, Italy; 2 Dipartimento Integrato Materno Infantile - Pediatria Generale e Specialistica - Pediatria ad Indirizzo Oncologico - Azienda Ospedaliera Universitaria, Policlinico di Modena, Via del Pozzo, 71, 41100 Modena, Italy; 3 Consorzio per Valutazioni Biologiche e Farmacologiche, Via Palestro 26, 27100 Pavia, Italy; 4 Dipartimento Integrato di Pediatria - Ospedale Pediatrico Bambino Gesù, Piazza Sant'Onofrio, 4 - 00165 Roma, Italy

## Abstract

**Background:**

The lack of availability of appropriate medicines for children is an extensive and well known problem. Paediatricians and Physicians who take care of the paediatric population are primarily exposed to cope with this negative situation very often as more than half of the children are prescribed off-label or unlicensed medicines.

**Discussion:**

Medicinal products used to treat this population should be subjected to ethical research of high quality and be explicitly authorised for use in children as it happens in adults. For that reason, and following the US experience, the European Paediatric Regulation has been amended in January 2007 by the European Commission. The objective of the Paediatric Regulation is to improve the development of high quality and ethically researched medicines for children aged 0 to 17 years, to facilitate the availability of information on the use of medicines for children, without subjecting children to unnecessary trials, or delaying the authorisation of medicines for use in adults.

**Summary:**

The Paediatric Regulation is dramatically changing the regulatory environment for paediatric medicines in Europe and is fuelling an increased number of clinical trials in the paediatric population. Nevertheless, there are some risks and pitfalls that need to be anticipated and controlled in order to ensure that children will ultimately benefit from this European initiative.

## Background

Children in Europe represent more than 20% of the European population, with about 100 million people aged less than 19 years.

The lack of specific drugs and labelling recommendation for the paediatric population is a long-standing worldwide problem and evidence-based prescribing for children is compromised by lack of satisfactory data on many drugs: 50% to 75% of medicines used in children have not been studied adequately in the paediatric population [1-4].

Although before a medicinal product for human use is marketed, it has to have undergone extensive studies to ensure its quality, safety and efficacy for use in the target population, many drugs given to children have not been tested in this specific population at all [5]. The barriers to undertaking proper research on children's drugs development are long standing and include: the cost of studies compared with the size of the potential market which implies that conducting research on medicines for children is often financially unrewarding for the pharmaceutical companies; difficulties in trial design (i.e., small numbers of eligible patients and lack of appropriate age-matched controls); time taken to complete studies in children as compared in adults; long approval processes; unique and complex ethical issues surrounding research on children; assessment of risk-benefit in those who cannot provide consent for themselves. Paediatric clinical trials of new drugs are often started many years after the drugs were tested in adults and involve testing at arbitrary doses and schedules on the basis of scaled down versions of those used in adults. By this time the drugs are already off-patent and the financial incentive for the pharmaceutical company to be involved in this phase of development will have lapsed [6]. A drug can be licensed for use as a medicine with indications, doses and side-effect warnings just if evidence for the safety, efficacy and acceptable risk/benefit exists. Nevertheless, prescribing can also be unlicensed (i.e. the medicine is given as a liquid whereas the licence is for a tablet) or off-label, that means outside the terms of the licence (i.e. a different indication or different dose for a different age group). For oral drugs, a formulation suitable for very young children who are unable to swallow tablets or capsules is often unavailable. This unavailability means clinicians have had to improvise the administration of such drugs to these children, with unknown pharmacokinetic consequences. More than half of prescriptions for children overall, more than 80% for children with cancer and about 90% of prescriptions for neonates are for medicines which have not been licensed for that use [6-9]. Although there may be concerns in conducting trials in the paediatric population, this has to be balanced by the ethical issues related to giving medicines to a population in which they have not been tested and therefore their effects, positive or negative, are unknown. Paediatricians very often face the dilemma of whether it is more unethical to prescribe a drug that has not been studied for children or to deny a potential useful treatment to a sick child. The result is that Physicians frequently prescribe marketed medicines to children on the basis of clinical practice and medical knowledge, without any chance to address questions concerning the risk of underdosing or overdosing the substance as well any appropriate consideration about early and even more late side effects.

Market forces alone have proven insufficient to stimulate adequate research aimed at specific development and authorisation of medicinal products for the paediatric population. Problems resulting from the lack of suitably adapted medicinal products for children include inadequate dosage information which leads to increased risks of adverse reactions including death, ineffective treatment through under-dosage, non-availability to the paediatric population of therapeutic advances, suitable formulations and routes of administration, as well as use of master or officinal formulations to treat the paediatric population which may be of poor quality. Children of different ages are vulnerable groups with specific developmental, physiological and psychological differences from adults, which make age and development related research of medicines particularly important. In terms of both public health and ethics, it is clearly preferable to test medicines in children, in a safe and controlled paediatric clinical trial environment, where the individual child is protected, than to prescribe and use medicines in children never designed and evaluated for this particular use.

All these aspects have represented the ground for the development of the Paediatric Regulation (EC) N° 1901/2006 [10] entered into force on 26^th ^January 2007. This regulation applies, in its present form, in each member state on the very day of its publication, without any need for implementation in national laws, as opposed to a European Directive, such as the Clinical Trial Directive (European Union Directive 2001/20/EC) [11].

Adoption of the Paediatric Regulation followed years of preparation. The discussions around the drafts were mostly based on the experience of the US that implemented similar but not identical measures in 1997, providing incentives for the development of paediatric medicines [12].

In December 2000, the European Parliament voted a resolution addressing the need for better medicines for children in Europe and asking the Commission to prepare a new regulation. Members of the European Parliament considered that there was indeed a health issue to be addressed and resolved at the EU level. Six years later, the EU regulation was published. Through the establishment of a framework of rewards, incentives, and obligations for pharmaceutical companies, the legislation aims to encourage the development of medicines appropriately tested, authorized, and formulated for use in children. This European law is going to impact significantly access to new drugs for children. By considerably changing the landscape of drug development for children, the law will provide an opportunity to make further progress in the cure and quality of cure of children. However, there are some risks and pitfalls that need to be anticipated and controlled in order to ensure that children will ultimately benefit from the European initiative in the most broaden way.

## Discussion

### The Paediatric Regulation

The objective of the Paediatric Regulation is to improve the health of children in Europe by facilitating the development and availability of medicines for children aged 0 to 17 years. The first aim of the Paediatric Regulation is to make medicines available for children that means changing the way in which medicines are developed. Increasing the development of medicines for children is to be reached by ensuring that they are subject to high quality research, to avoid, at the same time, unnecessary clinical trials in children and not delaying the authorization of medicines for the adult population. In particular, developing medicines for children should be performed in the relevant population subsets including the neonate as well as children with cancer, which is still the first cause of death in the paediatric age due to diseases. These particular subsets are the most exposed to medicines that have neither been tested nor fully assessed. Developing medicines in children should also consider ethical aspects as the vulnerability of children. This is why there is the possibility of waivers of the development of a medicinal product for children when the medicine is potentially harmful or ineffective, when its development would not bring any significant therapeutic benefit to children, and obviously, when the disease to be treated does not occur in children.

The new key element of the Regulation is the early involvement of a pharmaceutical company in the research and development programme of a medicinal product by the requirement to receive an agreement on the proposed process for a new medicinal product, the so called Paediatric Investigational Plan (PIP).

This contains two elements either to get a waiver or an agreement on the clinical trials, and, if necessary, a deferral in children to be included in the development programme (PIP). This is in order to ensure that the necessary data are generated determining the conditions in which a drug may be authorised to treat the paediatric population. The timing and the measures proposed to assess quality, safety and efficacy in all subsets of the paediatric population that may be concerned shall be presented in a PIP. Furthermore, any measures to adapt the formulation of the medicinal product for its use in the paediatric population shall be included.

Pharmaceutical companies are asked to prepare and submit a PIP at the end of phase I or II pharmacological studies. Once approved by the European Medicines Agency (EMA), the PIP should be undertaken to generate paediatric data that will eventually be submitted in the application.

Every PIP presented by industry must be submitted to the Paediatric Committee (PDCO) of the European Medicines Agency (EMA), a new Committee composed by paediatric experts from all over Europe and created as stated in the Regulation.

The PDCO has been operational with 27 members, even before finalisation of the appointment by the European Commission of the further 6 members representing healthcare professionals and patients' associations. The PDCO assesses paediatric investigation plans and provides advice. All information is made public on the EMA website. In addition, specific measures have been or are in the process of being implemented by the EMA such as an inventory of paediatric needs, the creation of a network of paediatric networks to facilitate the conduct of high-quality paediatric clinical studies and the preparation of guidelines.

Since its establishment in July 2007 the PDCO has assessed a large numbers of procedures: by September 2009, 564 validated PIPs/waivers have been submitted by pharmaceutical companies covering nearly 870 indications. PDCO approved 294 applications (i.e. relating to full waiver, and PIP, including potential deferral) covering more than 20 therapeutic areas (Table 1).

**Table 1:** PIPs/waivers approved by PDCO on September 2009.

Therapeutic area	PIPs	Waivers
Anaesthesiology		1(1RW)

Bone disease		1

Cardiology	1	5

Cardiology/endocrinology/metabolism		1

Cardiovascular diseases	18	16

Dermatology	6	2

Diagnostic and other	1	1

Endocrinology	1	

Endocrinology and metabolism	6	5

Endocrinology/gynaecology/	3	3

Endocrinology/gynaecology/fertility/metabolism	18	16

Gastroenterology	1	

Gastroenterology Haemostaseology	6	

Gastroenterology Hepatology	6 (1RP)	2

Gastroenterology-Hepatology Dermatology Immunology-Rheumatology-Transplantation	1	

Gastroenterology-Hepatology/Immunology	1	

Gynaecology		1

Haemathology		3

Haemathology/haemostaseology		1

Hepatology	1	

Immunology	3	1

Immunology/rheumatology/transplantation	10	1

Infectious diseases	24	1

Metabolism	5	

Nephrology		1

Neonatology - paediatric intensive care	1	

Neurology	9	5

Nutrition	2	

Oncology	16	13

Oncology - Endocrinology-Gynaecology-Fertility-Metabolism - Immunology-Rheumatology-Transplantation	1	

Ophthalmology	3	6

Oto-rhino-laryngology	1	

Oto-rhino-laryngology - Pneumology - Allergology - Dermatology	1	

Pain	9	7

Pain and neurology	1	

Pneumology	7	4

Pneumology - allergology	5	3 (2RW)

Psychiatry	3	1

Rheumatology		2

Urology		2

Uro-Nephrology	2	2

Vaccine	20	2

When needed, the PDCO has the power to ask the company for modifications or additional information at a later date; this especially because the PIP should be submitted at such an early point in the development process and some aspects will be preliminary. Once received the PIP, PDCO must consider whether or not any proposed studies will be of significant therapeutic benefit to the paediatric population. If not, the PDCO itself will impose a waiver from the requirement to provide data from a PIP (for lack of safety or efficacy or significant therapeutic benefit over existing treatments). Approval of the PIP by PDCO is necessary in order to gain both the marketing authorization and the financial benefits. If a PIP is not approved then a pharmaceutical company can resubmit it, and an additional appeal process exists through the European courts.

In addition, to the scrutiny of PIPs, the PDCO has several other tasks mandated by the regulation and it is especially contributing to other aspects of paediatric drug development such as guidance and reflection on of innovative methods of development.

In order to avoid unnecessary and unethically repetition of trials in children, paediatric development is addressed globally through a close cooperation with the US Food and Drug Administration (FDA) and other regions. Monthly teleconferences take place between the EMA and the FDA.

Considering that for the first time, companies will be required to study medicines in the paediatric population and develop age-appropriate formulations, the Regulation includes a system of rewards and incentives, to stimulate paediatric drugs development and to reward the industry for conducting the obligatory investigation programmes.

When an agreed PIP is completed and all the information has been submitted to the regulatory authorities, the medicinal product will be granted an extra 6 months patent protection (extension of the duration of its Supplementary Protection Certificate [SPC]), which represents the reward to pharmaceutical companies. This extension will be granted whether or not the data support a paediatric indication. A compound is usually protected for 7 years during which no generic compound can be marketed and the pharmaceutical company can thus enjoy returns on its investments. The 6 months extension is a substantial reward for most of the drugs since the extended market exclusivity represents significant benefits when the drug is sold throughout Europe.

For orphan medicinal products the incentive takes the form of an extra two years market exclusivity.

The Regulation also establishes a new type of marketing authorisation, called the paediatric use marketing authorisation (PUMA), intended to stimulate the development of off-patent products for use in the paediatric population. Only medicines that are intended solely for use in children will be eligible for a PUMA. Indeed, there is still a need for additional paediatric information on off-patent medicines as most of these compounds are in the public domain, are still widely used daily in children of all age groups, have not been adequately tested in the paediatric population. The PUMA is the specific instrument created by the Regulation, which can be granted for off-patent medicine that provides additional information according to the needs identified by the EMA. The Paediatric Regulation includes provisions for funding of research into off-patent medicines. Public funding is necessary as off-patent medicines are of little commercial interest for pharmaceutical companies. The PDCO has recently updated a list of priorities that indicates the areas and products that require paediatric development and should have potential for funding by the European Commission (Directorate-General Research, Brussels, Belgium). Six projects have obtained funding in the first exercise in 2007 [13]. Also the 7th European Framework Programme launched specific calls for proposals to support studies on these old drugs which are unlikely to be funded by large pharmaceutical companies. Public and public/private consortia are currently funded to study drugs in agreement with the priority list established by the EMA. Although they are relatively weak so far it is hoped that the incentives associated with a PUMA will encourage the development of new paediatric formulations for these old products.

The second main pillar of the Paediatric Regulation is to increase paediatric research. Clinical trials in the paediatric population require specific expertise, in some cases specific methodology and specific facilities, and should be carried out by appropriately trained investigators. Two main approaches are being implemented in order to facilitate this aspect: the creation of an European Network of Paediatric Research, and research funding. The EMA is responsible for establishing a network of existing networks, centres and investigators of paediatric research. The strategy for the establishment of the European Network has now been adopted and published [14,15]. The network's objectives are to coordinate studies relating to paediatric medicinal products, to build up the necessary scientific and administrative competences at European level, in order to avoid duplication of studies and testing in children.

EMA is supporting the development of this strategy by providing free paediatric scientific advice from, information tools (i.e., an inventory of therapeutic needs, information on new product labelling requirements, a public database of clinical trials), enhanced safety monitoring for marketed products concerning the obligation to include long-term follow-up of adverse drug reactions (ADRs) and the requirement for post marketing data for pharmacovigilance.

The benefits of a European Network of paediatric research include technical and/or administrative competences in the performance of paediatric clinical trials through effective collaboration. They also include avoiding duplication of work and efforts, making the use of facilities more efficient and profitable, developing common methods of working with special attention to quality assurance. Additional benefits are the facilitation of recruitment of patients, and avoiding unnecessary studies in children. The EU network should serve as a tool for industry to perform trials with children in keeping with the PIP.

The third aim of Regulation is transparency and information. Through increased availability of information, the safe and effective use of medicinal products for children can be increased so promoting public health. In addition, availability of this information will help prevent the duplication of studies in children avoiding unnecessary studies. One of the measures is making all paediatric trials included in the European database (EudraCT) accessible to the public both for protocol and results-related information. The increase of transparency in respect to clinical trials in children in all phases of the progress, beginning from the planning and recruiting of patients to the on-going and finalised studies, is another target of the Regulation. All decisions of the EMA on PIPs, deferrals or waivers of the paediatric development have to be made public and are routinely published on EMA website [16]. Moreover where authorisation is granted, the results of all those paediatric studies, any waivers or deferral, shall be included in the Summary of Product Characteristic and, if appropriate, in the Patient Leaflet of the medicinal product, whether or not all the paediatric indications concerned were approved by the competent authority.

### Considerations and expectations

Thanks to the Paediatric Regulation, safety and efficacy studies in the paediatric population have become mandatory for any drug likely to be used to treat children for which a new marketing authorisation or marketing authorisation variation is requested, as well as incentives for off-patent drugs. The regulation also requires that data for long-term follow-up of ADRs are included in any clinical trials that take place. Marketing authorisation variations are especially relevant to many drugs that are only authorised for use in adults, but which are frequently used in children.

This regulation is a remarkable step forward, because for the first time in Europe it is a regulation that is provided by law and provides direct economic support for paediatric clinical trials and indirect support for pharmaceutical industries.

The effect of the new regulation is expected to stimulate high-quality research and provide robust information on paediatric drugs to increase the availability of such drugs to children. This regulation aims to keep ineffective treatment, incorrect dosing and ADRs to a minimum; to reduce hospitalisations and deaths; to improve quality of life; and to provide economic benefits. The expectation of this regulation is that it will provide the paediatric population with safe access to older drugs and early access to newer, safer, and more targeted treatments.

The implementation of the European Regulation on medicines for paediatric use means also that in both the Europe and the USA, there is now a similar approach to paediatric drug development. Both require pharmaceutical companies to consider the needs of children and to carry out appropriate studies, and both provide financial rewards to companies who comply. It would be reasonable to assume that manufacturers will adopt strategies to ensure that new medications will pass both sets of regulations in parallel, to minimize time and maximize profits.

An integrated overview procedure is also included in the new European Regulation, with the European Commission charged with writing a report on the positive and negative aspects of its implementation, including the economic and public health effects. At the time being it is not always possible to foresee how a new piece of legislation will affect a marketplace, nevertheless it appears that Europe has learnt from the initial US experience, finally giving the development of drugs for children a legal status. This should allow greater and safer drug development for this vulnerable previously neglected population.

## Summary

To tackle the lack of specific drugs developed for paediatric population a new Paediatric Regulation entered into force in the European Union on 26^th ^January 2007. The objective of the Paediatric Regulation is to improve the development of medicines of high quality and ethically researched for children aged 0 to 17 years, to improve the availability of information on the use of medicines for children, without subjecting children to unnecessary trials or delaying the authorisation of medicines for use in adults.

The Regulation has provided the establishment in Europe of a mandatory Paediatric Investigation Plan (PIP) which is legally binding for manufacturers willing to seek marketing authorisation with a systems of incentives and rewards, and the establishment of a permanent Paediatric Committee at the EMA. The Regulation also establishes a new type of marketing authorisation, called the Paediatric Use Marketing Authorisation (PUMA), intended to stimulate the development of off-patent products for use in the paediatric population. The Regulation is also aimed to the creation of an European Network of paediatric research, and research funding.

The Paediatric Regulation is dramatically changing the regulatory environment for paediatric medicines in Europe and is fuelling an increased number of clinical trials in the paediatric population.

## List of abbreviations used

PIP: Paediatric Investigational Plan; PDCO: Paediatric Committee; EMA: European Medicines Agency; FDA: Food and Drug Administration; SPC: Supplementary Protection Certificate; PUMA: Paediatric Use Marketing Authorisation; ADRS: Adverse Drug Reactions; EUDRACT: European Clinical Trials database.

## Competing interests

All the authors declare that they have no competing interests as they have been described in the "manuscript sections for Debate Articles" of the IJP.

Authors are members of the Paediatric Committee (PDCO) since 2007 (Paolo Rossi, Francesca Rocchi) and since 2008 (Adriana Ceci, Paolo Paolucci). Paolo Rossi was previously member of the Paediatric Expert Group at EMEA, which preceded the institution of PDCO [Paediatric Regulation (EC) N° 1901/2006, entered into force on 26^th ^January 2007].

## Authors' contributions

All the Authors equally contributed to this article by providing substantial contributions to the analysis, understanding and interpretation of the new regulation. They have been equally involved in drafting the manuscript or revising it critically. Finally, all authors approved the final content manuscript.
